# Long-Term Fate Mapping Using Conditional Lentiviral Vectors Reveals a Continuous Contribution of Radial Glia-Like Cells to Adult Hippocampal Neurogenesis in Mice

**DOI:** 10.1371/journal.pone.0143772

**Published:** 2015-11-23

**Authors:** Sarah-Ann Aelvoet, Jesus Pascual-Brazo, Sarah Libbrecht, Veerle Reumers, Rik Gijsbers, Chris Van den Haute, Veerle Baekelandt

**Affiliations:** 1 Laboratory for Neurobiology and Gene Therapy, Department of Neurosciences, KU Leuven, Leuven, Flanders, Belgium; 2 Leuven Viral Vector Core, KU Leuven, Leuven, Flanders, Belgium; Charité-Universitätsmedizin Berlin, GERMANY

## Abstract

Newborn neurons are generated throughout life in two neurogenic regions, the subventricular zone and the hippocampal dentate gyrus. Stimulation of adult neurogenesis is considered as an attractive endogenous repair mechanism to treat different neurological disorders. Although tremendous progress has been made in our understanding of adult hippocampal neurogenesis, important questions remain unanswered, regarding the identity and the behavior of neural stem cells in the dentate gyrus. We previously showed that conditional Cre-Flex lentiviral vectors can be used to label neural stem cells in the subventricular zone and to track the migration of their progeny with non-invasive bioluminescence imaging. Here, we applied these Cre-Flex lentiviral vectors to study neurogenesis in the dentate gyrus with bioluminescence imaging and histological techniques. Stereotactic injection of the Cre-Flex vectors into the dentate gyrus of transgenic Nestin-Cre mice resulted in specific labeling of the nestin-positive neural stem cells. The labeled cell population could be detected with bioluminescence imaging until 9 months post injection, but no significant increase in the number of labeled cells over time was observed with this imaging technique. Nevertheless, the specific labeling of the nestin-positive neural stem cells, combined with histological analysis at different time points, allowed detailed analysis of their neurogenic potential. This long-term fate mapping revealed that a stable pool of labeled nestin-positive neural stem cells continuously contributes to the generation of newborn neurons in the mouse brain until 9 months post injection. In conclusion, the Cre-Flex technology is a valuable tool to address remaining questions regarding neural stem cell identity and behavior in the dentate gyrus.

## Introduction

Since its controversial discovery in the 1960s, it has now been well established that adult neurogenesis occurs in mammals, including humans, throughout the entire lifespan [1–7]. The continuous generation of new functional neurons occurs in two distinct regions: the subventricular zone (SVZ) lining the ventricle wall and the hippocampal dentate gyrus (DG). The remaining plasticity of the adult mammalian brain has fueled the idea of boosting this endogenous phenomenon for repair purposes in neurological disorders and brain injuries. However, although tremendous progress has been made in deciphering different aspects of adult neurogenesis, from the identity of involved cells to different regulatory mechanisms [8,9] and eventually its functional relevance [10–14], there are still significant questions that need to be addressed before one can use adult neurogenesis for therapeutic purposes [15].

**Fig 1:**
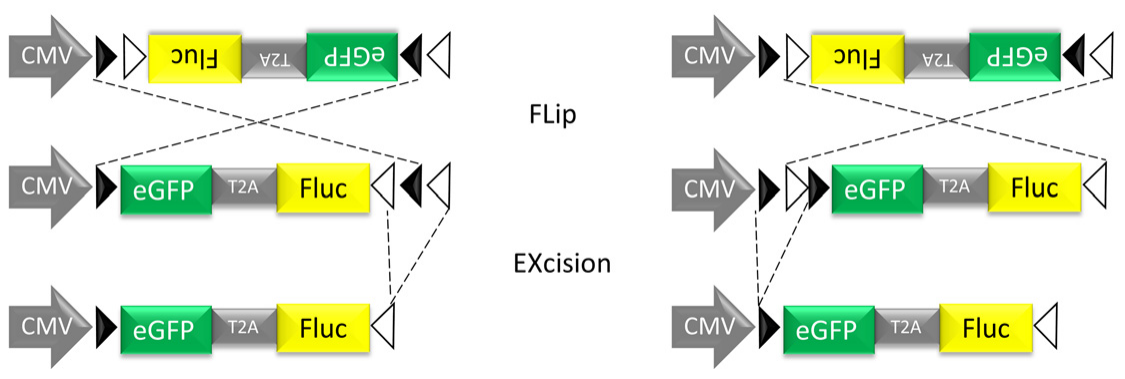
Schematic representation of the conditional Cre-Flex LV. The cDNA cassette is flanked by one pair of WT loxP sites (closed arrowheads) and one pair of mutant loxm2 sites (open arrowheads). In the presence of Cre recombinase, the DNA sequence between opposing sites is inversed (Flip), resulting in the positioning of two homotypic sites in the same orientation. The DNA sequence that is flanked by similarly oriented sites is excised (Excision). Cre-mediated inversion can start at the loxP or the loxm2 sites, but will always result in the same final product after Cre-mediated excision. The end product is an inverted DNA sequence, flanked by two heterotypic sites that cannot recombine with one another thereby preventing further inversions.

As in any field of science, progress in recognizing the existence and the key features of adult neurogenesis has been, in part, the result of a multidisciplinary and technological development. Nucleotide analogs such as 5-bromo-2'-deoxyuridine (BrdU), were the key players in the discovery of adult neurogenesis and are still widely used [1]. However, their use comes with different caveats like nonspecific incorporation into damaged DNA undergoing repair, dilution of the BrdU label after several rounds of cell division and the fact that experimental conditions might affect BrdU uptake. An alternative way of targeting proliferating cells is by local injection of retroviral vectors, which lack nuclear import mechanisms and thus rely on cell division in order to allow viral integration. Retroviral vectors have proven to be an important tool to morphologically characterize newborn cells through the expression of fluorescent proteins [16], to perform fate mapping of newborn neurons [17] as well as to manipulate gene expression using both gain- and loss-of-function strategies [18,19]. Electrophysiological characterization after retroviral transduction provided strong evidence that newborn neurons are functional and synaptically integrated [16]. However, as with the application of nucleotide analogs, the use of retroviral vectors has the disadvantage that they are not effective in labeling the true neural stem cells (NSCs), which are most of the time in a quiescent non-dividing state [20]. Because of the fact that they are biased towards labeling neurogenic rapidly dividing precursors, the use of these methodologies might even lead to the underestimation of glial or other alternative fate choices [21]. Lentiviral (LV) vectors also target the quiescent NSCs, since these vectors stably integrate in both dividing and non-dividing cells, thereby being an ideal tool for efficient and long-term *in vivo* labeling of NSCs in the SVZ [20,22]. However, LV vectors do not exclusively transduce NSCs, but also mature neurons and astrocytes at the site of injection. To obtain specific marking of a subset of cells, here NSCs, we developed conditional Cre-Flex LV vectors (Fig 1)[23]. Injection of these Cre-Flex LV vectors into the SVZ of transgenic Nestin-Cre mice resulted in specific labeling of the SVZ NSCs and eventually their progeny.

Another way to study adult neurogenesis is by non-invasive imaging in individual animals over time [24–30]. Bioluminescence imaging (BLI) is a simple, relatively inexpensive and sensitive imaging technology that allows non-invasive evaluation of a molecular process in the same group of animals over time [31–34]. BLI is based on luciferases, enzymes that generate visible light in living cells and organisms upon oxidizing a substrate. The most popular luciferase, firefly luciferase (Fluc), derived from the North American firefly *Photinus pyralis*, oxidizes a substrate called D-luciferin, a process that results in the production of light with a peak emission wavelength (λ_max_) of 612 nm. We and others previously showed the feasibility to efficiently and stably introduce Fluc in the NSCs by stereotactic injection of constitutive LV vectors into the SVZ, which allowed to monitor the migration of NSCs and their progeny towards the olfactory bulb (OB) with BLI [35,36]. More recently, we applied the conditional Cre-Flex LV technology to specifically label the NSCs of the SVZ in Nestin-Cre mice, that enabled us to monitor the neurogenic response in a stroke model, demonstrating that nestin^+^ NSCs originating from the SVZ respond to stroke injury by increased proliferation and migration of their progeny towards the infarct region [23].

To date, the possibility of using BLI to study adult hippocampal neurogenesis has not been investigated yet. In addition, conditional LV vectors have not been used before to perform fate mapping of neurogenesis in the DG. In the present study we verified whether the Cre-Flex LV technology can be applied to specifically label the NSCs of the DG and whether this would allow to monitor the hippocampal neurogenic process over time with BLI or histologically by fate mapping analysis at different time points.

## Materials & Methods

### Lentiviral vector construction and production

A conditional LV system based on the Cre/loxP mechanism, here referred to as Cre-Flex (Cre-mediated FLip-EXcision) was designed as described previously (Fig 1)[23]. Briefly, the Cre-Flex LV vectors carry a CMV-driven reporter cassette encoding eGFP and Fluc flanked by two pairs of mutually exclusive lox sites. The reporter cassette is activated after Cre recombination. The Cre-Flex LV vectors were generated and produced by the Leuven Viral Vector Core at the KU Leuven, essentially as described previously [37,38]. Viral titers were determined using p24 enzyme-linked immunosorbent assay (HIV-1 p24 ELISA kit, PerkinElmer, Milano, Italy) (unit: ng p24/mL).

### Stereotactic surgery

All animal experiments were carried out in accordance with the European Communities Council Directive of 24 November 1986 (86/609/EEC) and approved by the Bioethical Committee of the KU Leuven (permit number p020-2012). B6.Cg-Tg(Nes-cre)1Kln/J mice, here referred to as Nestin-Cre mice (stock nr 003771, Jackson Laboratory, Bar Harbor, ME, USA), [39]) and Nestin-CreER^T2^ mice [40] were back-crossed to albino C57BL/6-Tyr^c-2J^/J mice (stock nr 000058, Jackson Laboratory), creating white furred Nestin-Cre and Nestin-CreER^T2^ mice respectively, in a C57BL/6 genetic background. Albino C57BL/6, Nestin-Cre and Nestin-CreER^T2^ mice were 8 weeks old at time of stereotactic injection.

All the animals were housed under a 12 h light/ 12 h dark cycle with free access to food and water. Anesthesia was induced by intraperitoneal (i.p.). injection of a mixture of ketamine (75 mg/kg Ketalar, Pfizer, Brussels, Belgium) and medetomidine (1 mg/kg Domitor, Pfizer). The mice were placed in a stereotactic head frame (Stoelting, Wood Dale, IL, USA). A midline incision of the skin was made and a small hole drilled in the skull at the appropriate location, using bregma as reference. Coordinates to target the DG were anteroposterior (AP) -2.0 mm, mediolateral (ML) -1.5 mm relative to bregma, and dorsoventral (DV) -1.9 mm from the dural surface. Four μL of Cre-Flex LV vector was injected in the DG at a rate of 0.25 μL/min with a 30-gauge needle (VWR International, Haasrode, Belgium) on a 10 μL syringe (Hamilton, Bonaduz, GR, Switzerland). After the injection, the needle was left in place for an additional 5 min to allow diffusion before being slowly withdrawn from the brain. Anesthesia was reversed with an i.p. injection of atipamezol (0.5 mg/kg Antisedan, Pfizer). To induce Cre recombination in the Nestin-CreER^T2^ mice, tamoxifen was administered as previously described [40].

### *In vivo* bioluminescence imaging

The mice were imaged in an IVIS 100 system (PerkinElmer, Waltham, MA, USA). Anesthesia was performed in an induction chamber with 2% isoflurane (Halocarbon Products Corporation, River Edge, NJ, USA) in 100% oxygen at a flow rate of 1 L/min and maintained in the IVIS with a 1.5% mixture at 0.5 L/min. Since fur negatively influences BLI signals [31], the head of the mice was shaved before each imaging session. 126 mg/kg D-luciferin (Promega, dissolved in PBS (15 mg/mL)) was injected i.v. Immediately after injection, the mice were placed in the prone position in the IVIS and consecutive 1 min frames were acquired until the maximum signal, between 1 and 5 min after luciferin injection, was reached. The data are reported as the photon flux (p/s) from a 0.13 cm^2^ square region of interest.

### Perfusion and immunohistochemistry

Mice were deeply anesthetized by i.p. injection of pentobarbital (60 mg/kg, Nembutal, Ceva Santé Animale, Brussels, Belgium) and perfused transcardially with saline followed by ice-cold 4% paraformaldehyde in PBS. After post fixation overnight, 50 μm thick coronal brain sections were made with a vibratome (HM 650V, Microm, Walldorf, Germany). Immunohistochemistry was performed on every fifth section throughout the whole DG. Free-floating sections were pre-treated with 3% hydrogen peroxide (Chem-Lab, Zedelgem, Belgium) in PBS 0.1% Triton (PBS-T) for 10 min and incubated overnight with rabbit anti-eGFP (1:10.000, produced in-house [41]) or a rabbit anti-Cre recombinase (1:3000, [42]) in PBS-T with 10% swine serum (Dako). Biotinylated swine anti-rabbit secondary antibodies were used (1:300, Dako), followed by incubation with streptavidin-horseradish peroxidase complex (1:1000, Dako). Immunoreactivity was visualized using 3, 3-diaminobenzidine (DAB, 0.4 mg/ml, Sigma-Aldrich) as a chromogen. After a dehydration series, stained sections were mounted with DPX (Sigma-Aldrich) and visualized with a light microscope (Leica Microsystems, Wetzlar, Germany).

For the lineage tracing of eGFP^+^ cells in the DG, triple immunofluorescent stainings were performed. The sections were incubated overnight with the following primary antibodies: chicken anti-GFP (1:500, Aves Labs, Tigard, OR, USA), rabbit anti-glial fibrillary acidic protein (GFAP) (1:500, Dako), mouse anti-GFAP (1:200, BD Pharmingen, San Jose, CA, USA), goat anti-sex determining region Y-box 2 (Sox2) (1:200, Santa Cruz Biotechnology), goat anti-doublecortin (DCX) (1:200, Santa Cruz Biotechnology), mouse anti-neuronal nuclear antigen (NeuN) (1:200, Chemicon), rabbit anti-S100β (1:2500, Swant, Bellinzona, Switzerland) in PBS-T with 10% donkey serum. The next day, sections were incubated for 2 h in donkey anti-chicken–fluorescein isothiocyanate (FITC), donkey anti-goat-Alexa 555, donkey anti-mouse-Alexa 555, donkey anti-mouse-Alexa 647 and donkey anti-rabbit-Alexa 647. All secondary antibodies were diluted 1:400 and were purchased from Molecular Probes (Eugene, OR, USA), except donkey anti-chicken-FITC (Jackson Immunoresearch). Fluorescence was detected with a confocal microscope (FV1000, Olympus) with a 488 nm, a 561 nm and a 633 nm laser. Brightness, contrast, and background were adjusted equally per corresponding staining using the Fluoview software.

### Microscopic analysis and quantification

The total number of eGFP^+^ cells in the DG was quantified in a total of six sections, on every fifth coronal section starting at bregma -1.34 mm. Only eGFP^+^ cells in the SGZ and GCL were counted, excluding cells in the hilus and molecular layer. The quantification was performed with a light microscope (Leica Microsystems) via the optical fractionator method. A 100x100 μm^2^ grid was projected over each section and all eGFP^+^ cell bodies were counted at 40X magnification in a sampling volume of 100x100x14 μm^3^, omitting cells in the outermost focal plane. This approach resulted in counting 90–2000 eGFP^+^ cells in each DG yielding an average coefficient of error (Gundersen) of 0.054.

For the phenotypic analysis of eGFP^+^ cells in the DG, ratios of cells co-labeling with eGFP and GFAP, Sox2, DCX, NeuN or S100β were counted from confocal images of triple immunofluorescent sections. All eGFP^+^ in the SGZ and GCL in three sections per animal were analyzed at 40X magnification for co-expression with the different phenotypic markers. This approach resulted in the phenotypic analysis of 90–750 eGFP^+^ cells per animal. The absolute number of cells within each population was calculated by multiplying the population ratio by the absolute number of eGFP^+^ cells as determined by stereology.

### Statistical analysis

All statistical analyses were performed in Prism 5.0 (GraphPad Software). For comparisons at different time points, one-way ANOVA followed by a *post hoc* Tukey test to correct for multiple testing was used. In case of non-normality, the nonparametric equivalent (Kruskal–Wallis test) was chosen, followed by Dunn’s test. *p*-values are indicated as follows: *p < 0.05, **p < 0.01, ***p < 0.001.

## Results

### Cre-Flex LV vectors label a restricted cell population in the DG of Nestin-Cre mice, which can be detected with BLI

**Fig 2:**
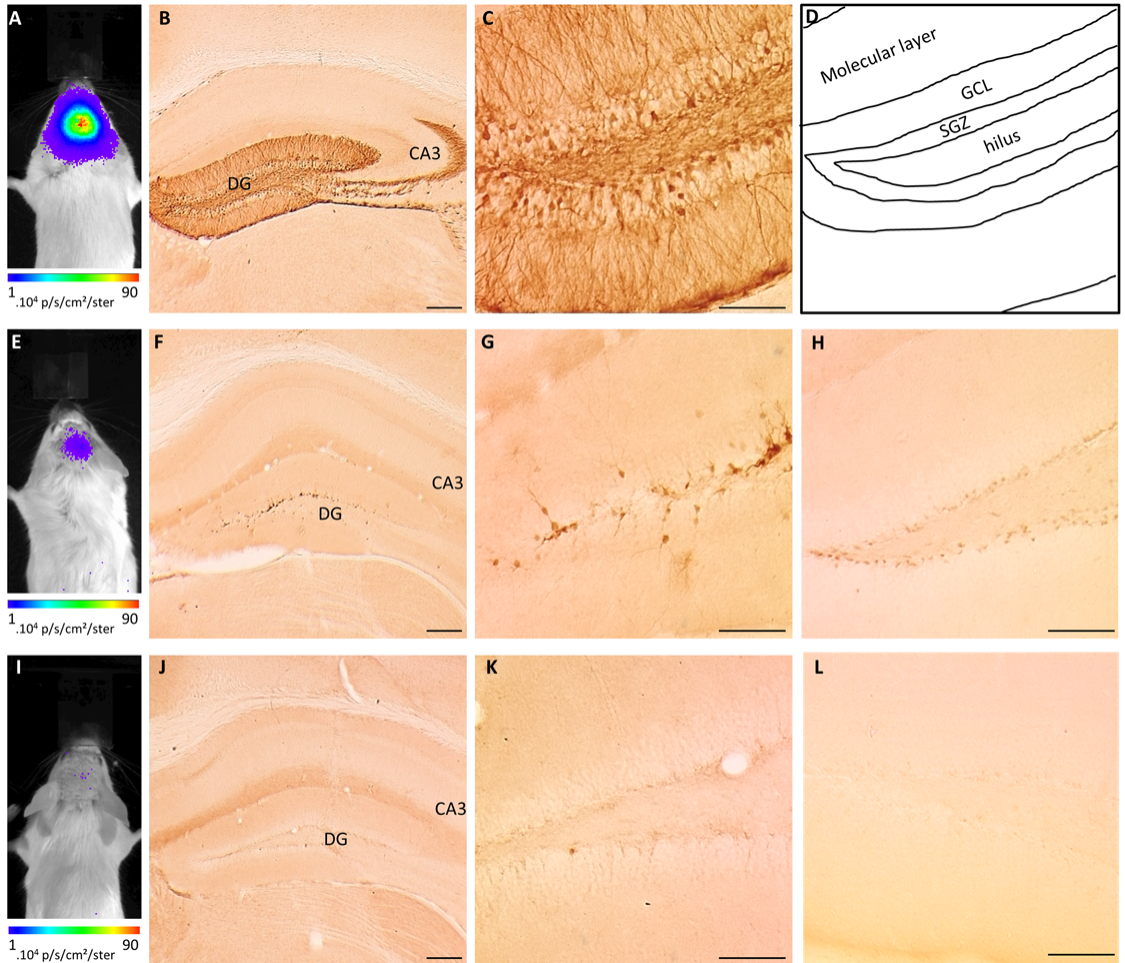
Cre-Flex LV vectors label a restricted cell population in the DG of Nestin-Cre mice. (**A-C**) WT mice were injected with a constitutive LV-eGFP-T2A-Fluc vector in the DG (n = 3). (**A**) Representative BLI image 6 days p.i. (**B-D**) Histological detection of eGFP^+^ cells across the DG including the hilus, the SGZ and the GCL (**C-D**). Labeled mature neurons in the GCL extend dendrites into the molecular layer and axons into the hilus that eventually project towards the CA3 (**B)**. (**D**) Schematic overview of the different regions of the DG. (**E-H**) Transgenic Nestin-Cre mice were injected with a LV-Cre-Flex><eGFP-T2A-Fluc vector in the DG (n = 4). (**E**) Representative BLI image 10 days p.i. (**F-G**) Histological detection of eGFP^+^ cells that mainly reside in the SGZ. (**H**) Histological detection of Cre^+^ cells in the SGZ of Nestin-Cre mice. (**I-L**) WT mice were injected with a LV-Cre-Flex><eGFP-T2A-Fluc vector in the DG (n = 3). (**I**) Representative BLI image 14 days p.i. (**J-K**) Histological detection of eGFP^+^ cells reveals very sparse labeling in the DG. (**L**) Absence of Cre^+^ cells in the DG of WT mice. Scale bar (**B,F,J**) = 250 μm; (**C,G,H,K,L**) = 100 μm.

The main purpose of this study was to monitor the neurogenic process in the adult hippocampus using conditional LV vectors. In a pilot experiment, we aimed to evaluate the labeling efficiency and specificity of the LV-Cre-Flex><eGFP-T2A-Fluc vector, further referred to as the Cre-Flex vector, in the DG compared to a constitutive LV vector. Different groups of animals were injected and both the BLI signals and pattern of labeled cells were analyzed. In a first group,WT micewere injected with a constitutive LV-eGFP-T2A-Fluc vector into the DG (n = 3). In these mice, a high BLI signal (8.3E^+05^±2.9E^+05^ p/s) emerged from the site of injection after six days (Fig 2A). Histological analysis revealed labeling of a broad population of cells, with eGFP^+^ cells across the DG, including the hilus, the subgranular zone (SGZ) and the granular cell layer (GCL) (Fig 2B–2D). Labeled mature neurons in the GCL extended dendrites into the molecular layer and axons into the hilus that eventually project towards the CA3 region (Fig 2B–2D). In parallel, transgenic Nestin-Cre mice were injected with the Cre-Flex LV vector (n = 4) (Fig 2E–2H). A 11-fold lower BLI signal (7.0E^+04^±3,6E^+04^ p/s) at 10 days post injection (p.i.) compared to WT mice injected with a constitutive LV emerged from the DG (Fig 2E). Histological analysis showed labeling of a more restricted population of cells, with eGFP^+^ cells mainly present in the SGZ (Fig 2F and 2G). The location of these eGFP^+^ cells is in line with detection of Cre^+^ cells that reside in the SGZ of Nestin-Cre mice (Fig 2H). As control, WT mice were injected with the Cre-Flex LV vector (n = 3) (Fig 2I–2L), in which no clear BLI spot was visible (1.5E^+04^±2.0E^+03^ p/s) at 14 days p.i. (Fig 2I) and only few eGFP^+^ cells were detected on histological analysis (Fig 2J and 2K). In addition, no Cre^+^ cells were detected in the DG (Fig 2L). Together, these data indicate that the Cre-Flex LV vector labels a restricted cell population in the DG of Nestin-Cre mice, which can be detected by BLI.

### Long-term BLI monitoring of Nestin-Cre and Nestin-CreER^T2^ mice

**Fig 3:**
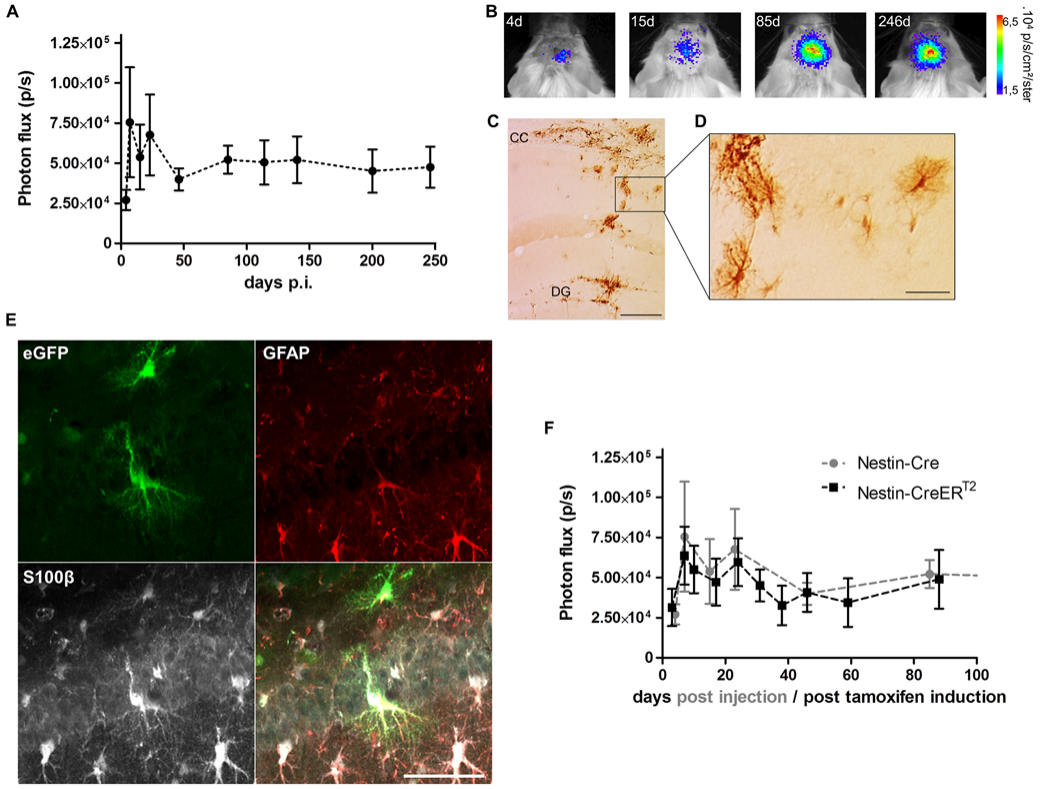
Long-term BLI of Nestin-Cre and Nestin-CreER^T2^ mice after injection of the Cre-Flex LV vector in the DG. (**A**) Nestin-Cre mice (n = 9) injected with the Cre-Flex LV vector were imaged with BLI on a regular basis until 9 months p.i. The BLI time course was initially characterized by fluctuations and high data variability, followed by stabilization of the signal after 45 days p.i. (**B**) Representative BLI scans of a Nestin-Cre mouse followed over time. (**C,D**) Four days after stereotactic injection, eGFP^+^ cells were not only detected in the DG, but also in the CC and injection tract where they displayed a typical stellar morphology as shown (**D**). (**E**) Triple immunofluorescent staining for eGFP, GFAP and S100β showed labeling of reactive astrocytes in the CC and injection tract. (**F**) Cre recombination was induced in Nestin-CreER^T2^ mice 3 weeks after injection of the Cre-Flex LV vector (n = 5), after which they were imaged regularly until 3 months post tamoxifen induction. The BLI kinetics of Nestin-CreER^T2^ were comparable to those of Nestin-Cre mice. Data of Nestin-Cre mice are the same as in (**A**). Scale bars: (**C)** = 250 μm; (**D,E**) = 50 μm.

To evaluate how the BLI signal evolves over time, the Cre-Flex LV vector was injected into the DG of a group of 8 week old Nestin-Cre mice (n = 9). Four days after injection, a BLI signal emerged from the site of injection that persisted until 9 months p.i., the latest time point monitored (Fig 3A and 3B). The initial course of the BLI measurements was characterized by an increasing but highly variable signal (Fig 3A). From 45 days p.i. until 9 months p.i, the BLI signal remained stable and was two-fold higher compared to 4 days p.i. Reactive astrocytes that upregulate nestin and thus Cre upon injury, induced by the stereotactic injection, might be the reason for the initial fluctuations of the BLI signal. To verify this hypothesis, another group of Nestin-Cre mice (n = 5) was injected with the Cre-Flex LV vector and perfused 4 days after injection. Histological analysis revealed eGFP^+^ cells in the DG, but eGFP^+^ cells with stellar morphology were also found in the injection tract and in the corpus callosum (CC) (Fig 3C and 3D). A triple immunofluorescent staining for eGFP, GFAP (expressed in NSCs and mature astrocytes) and S100β (only expressed in mature astrocytes), confirmed labeling of reactive astrocytes in the injection tract and in the CC (Fig 3E). However, labeled reactive astrocytes were absent at later time points p.i. (data not shown).

Nestin upregulation in reactive astrocytes peaks between 3 and 7 days after injury after which it declines to reach baseline level around 3 weeks after injury [43]. In order to prevent labeling of reactive astrocytes, the Cre-Flex LV was injected into the DG of inducible Nestin-CreER^T2^ mice (n = 5). Three weeks after stereotactic injection, Cre-mediated recombination was induced by tamoxifen administration. The mice were scanned with BLI until 3 months post tamoxifen induction. Remarkably, the BLI kinetics were characterized by initial fluctuations, similar to the Nestin-Cre mice, but less data variation was evident at these early time points (Fig 3F). These data, together with the fact that labeled reactive astrocytes could only be detected 4 days p.i. in the Nestin-Cre mice, indicate that the intitial fluctuations of the BLI signal are not due to reactive astrocytes.

### A time-dependent increase in the number of eGFP-labeled cells is not paralleled by a significant increase in BLI signal

**Fig 4:**
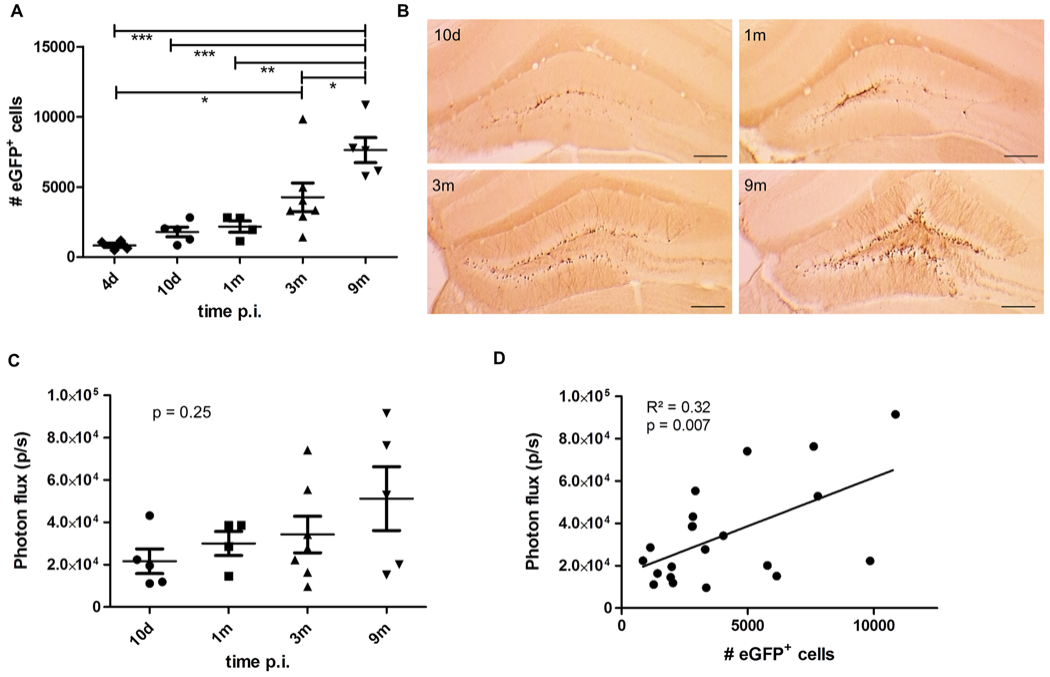
A time-dependent increase in number of eGFP-labeled cells is not paralleled by a significant increase in BLI signal. (**A**) Stereological quantifications of the number of eGFP^+^ cells in the DG of Nestin-Cre mice perfused at the indicated time points after injection of the LV-Cre-Flex vector. The number of eGFP^+^ cells continuously increased over time (*p* < 0.0001). At 9 months p.i., the number of eGFP^+^ cells was significantly higher compared to 4 days p.i. (p < 0.001), to 10 days p.i. (*p* < 0.001), to 1 month p.i. (*p* < 0.01) and to 3 months p.i. (*p* < 0.05). Raw data are shown in S1 Table. (**B**) Histological detection of eGFP^+^ cells at indicated time points. Scale bar = 250 μm. (**C**) Quantification of the BLI signal from the same animals depicted in (**A**). There was a trend towards a time-dependent increase of BLI signal but this was not significant (*p* = 0.25). (**D**) The number of eGFP^+^ cells per animal was correlated to the BLI signal of the same animal. A significant (Pearson correlation *p* = 0.007, n = 21) but weak (R^2^ = 0.32) correlation was evident.

**Fig 5:**
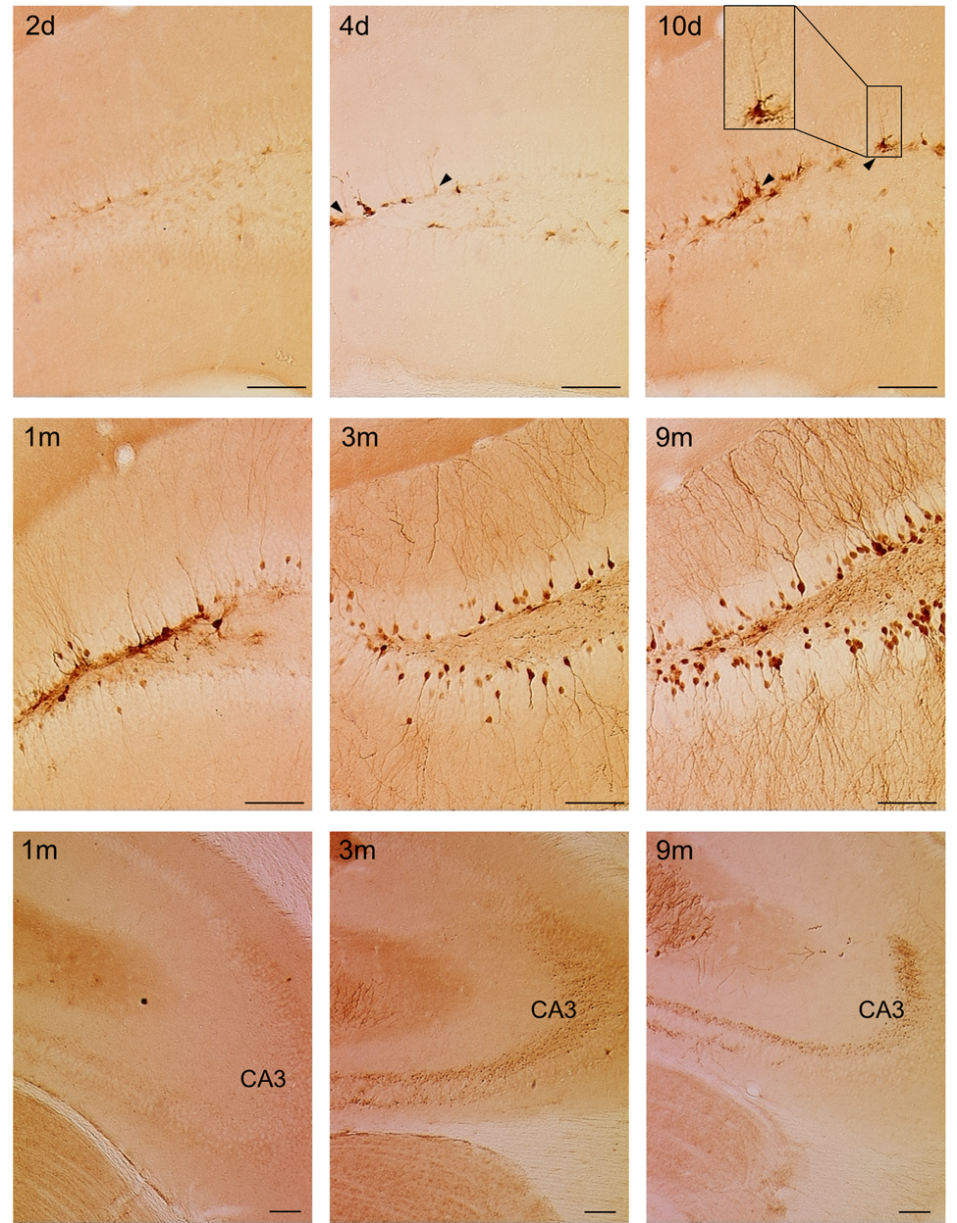
Dynamic changes in the pattern of labeled cells mirror the neurogenic cascade in the DG. Immunohistological detection of eGFP^+^ cells in the DG of Nestin-Cre mice at indicated time points after injection of the LV-Cre-Flex vector. At early time points, mainly cells in the SGZ are labeled (upper panel). Arrowheads indicate cells with a typical RGC morphology. At later time points, also cells in the GCL are labeled (middle panel). From 3 months p.i. onwards, labeled mossy fibers projecting towards the CA3 can be detected (lower panel). Scale bar = 100 μm.

To verify whether the number of labeled cells in the DG changes over time, extra groups of 8 week old Nestin-Cre mice were injected with the Cre-Flex LV vector and perfused at 2 days (n = 6), 10 days (n = 6), 1 month (n = 4) and 3 months p.i. (n = 4). Right before perfusion, the animals were imaged with BLI. The number of eGFP^+^ cells in the DG, including the SGZ and GCL, was determined via stereological quantifications. The number of eGFP^+^ cells continuously increased over time (One-way ANOVA combined with Tukey’s correction for multiple testing (*F*_(4,20)_ = 10.90), *p* < 0.0001), eventually reaching a 9.1-fold increase at 9 months p.i. compared to 4 days p.i. (*p* < 0.001) (Figs 4A, 4B and 5). At 2 days p.i., reliable stereological quantification could not be performed due to the low eGFP expression level (Fig 5 upper panel). In Nestin-CreER^T2^ mice, stereological quantifications revealed a 9-fold lower labeling efficiency 3 months post tamoxifen induction compared to Nestin-Cre mice (data not shown).

This time-dependent continuous increase in the number of eGFP^+^ cells in the DG of Nestin-Cre mice is in apparent contradiction with the stable BLI signal of the group of Nestin-Cre mice monitored until 9 months p.i. (Fig 3A). Closer evaluation of the BLI signals of the animals perfused at different time points p.i. showed a trend for a time-dependent increase in BLI signal but this was not significant (One-way ANOVA (*F*_(3,17)_ = 1.49), *p* = 0.25) (Fig 4C). When the number of eGFP^+^ cells in each individual animal was correlated with the value of the BLI signal measured for the same animal, a weak linear correlation was evident (Pearson correlation R^2^ = 0.32; *p* = 0.007; n = 21) (Fig 4D). These data indicate that BLI does not allow appropriate quantification of the number of labeled NSCs and their progeny in the DG over time.

### Dynamic changes in the pattern of labeled cells mirror the neurogenic cascade in the DG

Next to a time-dependent increase in the number of labeled cells, immunohistological detection of eGFP^+^ cells also revealed a time-dependent change in the labeling pattern (Figs 4B and 5). Two days p.i., there were few labeled cells with low eGFP expression levels (Fig 5 upper panel). At 4 and 10 days p.i., there was an increase in the expression of eGFP-labeled cells which were mainly present in the SGZ (Fig 5 upper panel). Some of the labeled cells extended a single apical process radially across the GCL towards the molecular layer, suggesting they were radial glia-like cells (RGCs), which are considered as the true NSCs (Fig 5 upper panel) [44]. From 1 month p.i. onwards, labeled cells were detected not only in the SGZ, but also in the GCL of the DG (Fig 5 middle panel). A substantial proportion of these cells showed the typical morphology of mature neurons with large dendritic trees branching into the molecular layer, which became more apparent at 3 and 9 months p.i. In addition, mossy fibers originating from these labeled mature neurons were detected in the hilus of the DG from 1 month p.i. onwards (Fig 5 middle panel). From 3 months p.i. onwards, the CA3 was densely innervated by these mossy fibers (Fig 5 lower panel). Together, these data indicate that at early time points after injection, the Cre-Flex LV vector mainly labeled progenitors residing in the SGZ. At later time points, labeled mature progeny that migrated into the GCL can also be detected. This indicates that the Cre-Flex LV vectors allow to monitor the neurogenic cascade in the DG of Nestin-Cre mice.

### Long-term fate mapping reveals a continuous contribution of RGCs to adult hippocampal neurogenesis

**Fig 6:**
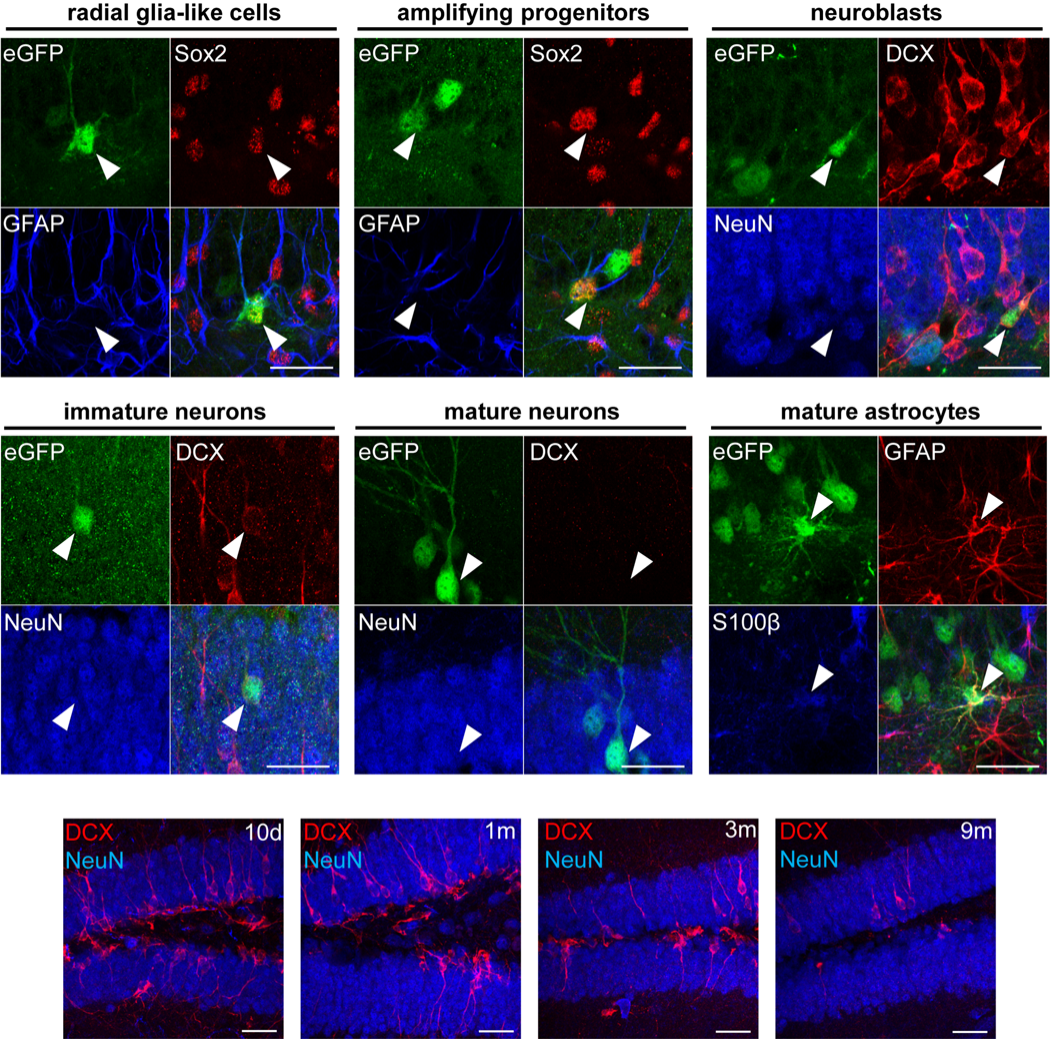
Phenotypical analysis of different labeled cell types in the DG. Upper and middle panel: Triple fluorescent stainings for eGFP/Sox2/GFAP, eGFP/DCX/NeuN and eGFP/GFAP/S100β followed by confocal analysis allowed to identify different labeled cell types as indicated. Lower panel: time-dependent decrease of DCX expression in the DG. Scale bars = 50 μm.

Since the Cre-Flex LV vectors label the NSCs of the DG and their progeny, they allow to determine the fate of this progeny using different phenotypical markers. Triple immunofluorescent stainings for eGFP/GFAP/Sox2 and subsequent confocal analysis revealed labeled GFAP^+^Sox2^+^ RGCs, the NSCs of the DG that reside in the SGZ and extend a single apical process radially across the GCL towards the molecular layer (Fig 6 upper panel). In addition, labeled GFAP^-^Sox2^+^ amplifying progenitors could be identified that also reside in the SGZ but lack an apical process (Fig 6 upper panel). A second triple staining for eGFP/DCX/NeuN allowed identification of 3 different cell types: a) labeled DCX^+^NeuN^-^ neuroblasts confined to the SGZ (Fig 6 upper panel), b) labeled DCX^+^NeuN^+^ immature neurons in the SGZ and GCL that carry a single apical process (Fig 6 middle panel) and c) labeled DCX^-^NeuN^+^ mature neurons in the GCL with an extensive dendritic tree (Fig 6 middle panel). Finally, a triple staining for eGFP/GFAP/S100β revealed GFAP^+^S100β^+^ mature astrocytes (Fig 6 middle panel).

**Fig 7:**
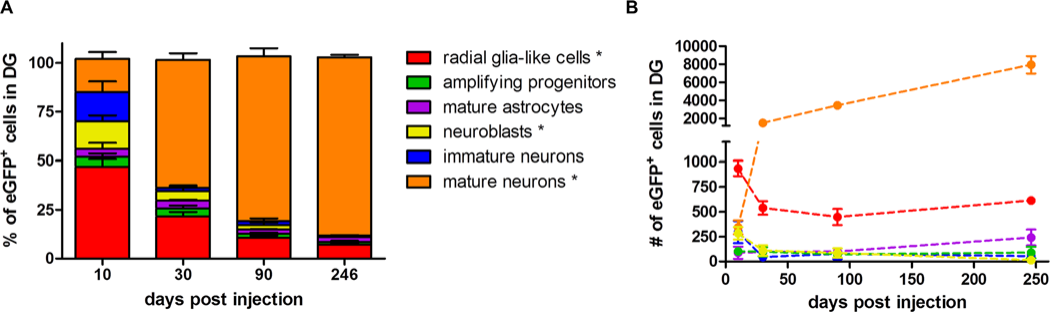
Long-term fate-mapping analysis in the DG of Nestin-Cre mice. (**A**) The proportion of different labeled cell types was determined at different time points p.i. The number of eGFP^+^ cells that was analyzed for co-localization with different phenotypical markers was 146±36 at 10 days p.i. (n = 4), 265±28 at 1 month p.i. (n = 3), 454±129 at 3 months p.i. (n = 3) and 647±48 at 9 months p.i. (n = 3). The proportion of eGFP^+^ RGCs (*p* = 0.01) and neuroblasts (*p* = 0.02) decreased over time, while the proportion of eGFP^+^ mature neurons increased (*p* = 0.01). Raw data are shown in S2 Table. (**B**) The absolute number of labeled cells within each population was calculated by multiplying the population ratio per animal by the absolute number of the eGFP^+^ cells as determined by stereology in the same animal. The number of eGFP^+^ RGCs initially decreased (*p* = 0.03) but remained constant after 1 month p.i. The number of eGFP^+^ neuroblasts decreased from 10 days to 9 months p.i. (*p* = 0.04). The number of eGFP^+^ mature neurons continuously increased over time (*p* = 0.01). Raw data are shown in S3 Table.

Fate-mapping analysis of the eGFP^+^ cells after injection of the Cre-Flex LV vector and subsequent quantification revealed that 10 days p.i., RGCs constituted the majority of eGFP^+^ cells (47%) (Fig 7A). The other labeled populations were mainly neuroblasts (14%), immature neurons (15%) and mature neurons (17%). Less than 5% of eGFP^+^ cells were either amplifying progenitors or mature astrocytes. Between 10 days and 9 months p.i., the proportion of labeled RGCs showed a 6.7 fold decrease and constituted 7% of the eGFP^+^ population at 9 months p.i (non-parametric Kruskal-Wallis test (*H*_(3)_ = 11.27), *p* = 0.01) (Fig 7A). The proportion of labeled neuroblasts decreased from 14% at 10 days p.i. to less than 1% at 9 months p.i. (Kruskal-Wallis test (*H*_(3)_ = 9.71), *p* = 0.02) (Fig 7A). Likewise, the proportion of immature neurons decreased over time but this was not significant (Kruskal-Wallis test (*H*_(3)_ = 7.37), *p* = 0.06). On the contrary, the proportion of labeled mature neurons increased over time, eventually representing 91% of the eGFP^+^ population at 9 months p.i. (Kruskal-Wallis test (*H*_(3)_ = 10.92), *p* = 0.01) (Fig 7A).

Since the total number of eGFP^+^ cells increased over time (Fig 4A), a decrease in the proportion of eGFP^+^ RGCs and neuroblasts might indicate that the absolute number of eGFP^+^ RGCs and neuroblasts remained either constant or decreased over time. On the other hand, the increase in the proportion of eGFP^+^ mature neurons automatically implicates an increase in the absolute number of eGFP^+^ mature neurons. In order to calculate the absolute number of labeled cells within each population, the population ratio per cell type per animal was multiplied by the absolute number of the eGFP^+^ cells as determined by stereology in the same animal (Fig 4A). There was a significant effect of time on the absolute number of labeled RGCs (Kruskal-Wallis test (*H*_(3)_ = 9.07), *p* = 0.03), neuroblasts (Kruskal-Wallis test (*H*_(3)_ = 8.57), *p* = 0.04) and mature neurons (Kruskal-Wallis test (*H*_(3)_ = 11.27), *p* = 0.01) (Fig 7B). Remarkably, the absolute number of labeled RGCs showed a 2-fold decrease between 10 days and 1 month p.i. but then remained constant until 9 months p.i (Fig 7B). The absolute number of labeled neuroblasts showed a continuous decrease over time, resulting in a 20-fold reduction between 10 days and 9 months p.i (Fig 7B). A trend towards a time-dependent increase in the number of labeled mature astrocytes was observed, but this was not significant (Kruskal-Wallis test (*H*_(3)_ = 3.27), *p* = 0.35). As expected, the absolute number of labeled mature neurons increased over time and at 9 months p.i. approximately 8000 new labeled neurons were added to the DG. The number of labeled mature neurons increased 4-fold between 10 days and 1 month p.i., followed by another 5-fold increase up to 9 months p.i., indicating a continuous increase in the number of newborn neurons (Fig 7B). Interestingly, a time-dependent decrease in total DCX^+^ expression between 10 days and 9 months p.i. was observed (Fig 6 lower panel). Although we did not quantify this phenomenon, it suggests a time-dependent decline of neurogenesis in the DG [45]. Together, these data demonstrate that from 1 month p.i. onwards, a stable population of labeled RGCs continuously contributes to adult hippocampal neurogenesis over time.

## Discussion

During the last two decades, tremendous progress has been made in understanding key features of adult hippocampal neurogenesis. However, there are still unresolved questions regarding NSC identity and heterogeneity, how these NSCs give rise to new neurons and eventually also the functional role and therapeutic potential of adult hippocampal neurogenesis. In order to address these different questions, an expansion of the available toolbox is necessary. In a first part of this study, we verified whether BLI can be used to non-invasively monitor adult hippocampal neurogenesis over time. In a second part of the study, we took advantage of specific labeling of nestin^+^ RGCs in the DG to perform long-term fate-mapping analysis.

To monitor adult hippocampal neurogenesis with BLI, we applied the Cre-Flex LV technology, which previously allowed us to monitor neurogenesis in the SVZ [23]. Injection of the Cre-Flex LV vectors in the DG of Nestin-Cre mice resulted in labeling of a restricted population of progenitors and eventually their progeny that could be monitored with BLI until 9 months p.i. However, the long-term monitoring revealed some unexpected BLI kinetics. First, early after stereotactic injection, the course of the BLI signal was characterized by high data variability, which might result from labeling of reactive astrocytes that upregulate nestin upon injury [46]. We detected labeled reactive astrocytes in the injection tract and CC at 4 days p.i, which might contribute to higher BLI signals due to their more superficial location. In order to prevent labeling of reactive astrocytes, the Cre-Flex LV vector was injected in inducible Nestin-CreER^T2^ mice and Cre recombination was only induced 3 weeks after stereotactic injection. Surprisingly, the BLI signal showed a similar kinetic profile as was observed in Nestin-Cre mice excluding the reactive astrocytes as the source of the fluctuating BLI signal, but less data variability was evident. However, the labeling efficiency in Nestin-CreER^T2^ mice was 9-fold lower compared to Nestin-Cre mice, making it more challenging to detect dynamic changes in neurogenesis. On the other hand, this low labeling efficiency might be interesting to study adult neurogenesis at the clonal level instead of population level [21].

Second, the expansion of the labeled cell population over time was not translated into a significant increase in BLI signal. In contrast, we previously successfully used BLI to monitor the migration of NSC progeny from the SVZ towards the OB [35]. In the OB, both the BLI signal and the number of labeled cells increased until 3 months p.i. A major difference between the SVZ and DG is the distance and direction of migration of the NSC progeny. In the SVZ, the progeny migrates several millimeters away from its origin towards the OB. The rostrocaudal spatial difference between the SVZ and OB eventually results in two distinct BLI signals, with the BLI signal of the OB only reflecting addition and survival of newborn mature neurons. As a result, a strong correlation (R² = 0.85) between the number of labeled cells and BLI signal was observed in the OB [35]. On the contrary, in the DG, the newborn progeny only migrates a few micrometers in the dorsoventral direction [47]. As a result, all the labeled cell populations, ranging from NSCs to mature neurons are combined into one BLI signal. Together with the fact that the events of proliferation, apoptosis and survival are reflected in one BLI signal, we suggest that this underlies the poor correlation between the number of labeled cells and BLI signal (R^2^ = 0.32) in the DG. Dual-color BLI might be useful in order to distinguish different labeled cell populations [48]. However, luciferase pairs of higher photon emission in the red and far-red range first need to be developed in order to allow dual-color BLI in the brain. In conclusion, BLI allowed to detect the population of labeled cells in the DG, but did not allow to monitor the continuous generation of newborn neurons in the DG.

Although BLI could not be applied to monitor neurogenesis in the DG, we took advantage of the eGFP reporter protein also present in the Cre-Flex LV construct to evaluate hippocampal neurogenesis with histological techniques. Early after injection of the Cre-Flex LV vector, the majority of labeled cells were mainly RGCs, as evidenced by their location in the SGZ, their morphology and their phenotypic identity. This indicates that the Cre-Flex LV vectors specifically labeled the nestin^+^ population of the DG, which is in line with the specific labeling of NSCs after injection of LV-Cre-Flex vectors in the SVZ [23].

A second important finding was the continuous increase in the number of eGFP^+^ cells in the DG, which confirms that adult hippocampal neurogenesis in rodents is additive [47,49]. Different research groups already performed fate-mapping studies using different inducible Nestin-CreER^T2^ mice crossed with a reporter line, which led to conflicting results [21,40,45,50,51]. The continuous accumulation of eGFP^+^ cells we observed is in line with a study from Dranovsky *et al*. who evaluated the neurogenic contribution of the nestin^+^ population, showing a continuous increase in the number of labeled cells until 12 months after tamoxifen administration [45]. In addition, our fate-mapping analysis revealed that, after an initial decrease between 10 days and 1 month p.i., the number of labeled RGCs remained constant until 9 months p.i. This is in line with a report from Bonaguidi *et al*. who studied the nestin^+^ RGCs at the clonal level, revealing their capacity for long-term maintenance via symmetric divisions [21]. On the other hand, Encinas *et al*. showed that the nestin^+^ NSC population divides up to three times in rapid succession, eventually giving rise to new neurons, but meanwhile the nestin^+^ NSCs themselves ultimately exhaust due to astrocytic differentiation [50]. In the present study, the number of labeled RGCs showed a 2-fold decrease between 10 days and 1 month p.i. Together with the trend towards an increase in number of labeled astrocytes over time, this might indicate a partial disappearance and astrocytic differentiation of NSCs. Recently, the neurogenic potential of nestin^+^ RGCs was compared to GLAST^+^ (Glutamate Aspartate Transporter) RGCs, by comparing Nestin-CreER^T2^:R26R-YFP to GLAST-CreER^T2^:R26R-YFP mice [51]. In the Nestin-CreER^T2^:R26R-YFP mice, the number of YFP^+^ cells only transiently increased until 65 days after tamoxifen administration, followed by a stabilization. In the GLAST-CreER^T2^:R26R-YFP mice, however, the number of YFP^+^ cells continuously increased until 6 months after tamoxifen administration. The authors hypothesize that the GLAST^+^ RGCs represent ‘long-term’ largely dormant RGCs that, upon activation, become ‘short term’ nestin^+^ RGCs that divide a limited number of times before losing their proliferative potential. However, in our study we clearly demonstrate a continuous expansion of the eGFP^+^ cell pool, which is mainly reflected by a continuous increase in the number of labeled mature neurons. Together with the presence of a substantial number of labeled nestin^+^ RGCs at 9 months p.i., our results suggest that at least some labeled nestin^+^ RGCs are a continuous source for adult hippocampal neurogenesis.

A substantial amount of published fate-mapping studies evaluate adult neurogenesis by quantifying the proportion of different labeled cell types over time, without taking into account possible changes in the total number of labeled cells. In our study, evaluating the proportion of RGCs would have led to the conclusion that the labeled RGCs continuously deplete over time, reaching a 6.7-fold reduction after 9 months. However, when taking into account the stereological quantifications of the total number of eGFP^+^ cells, the number of labeled RGCs only decreased 1.7-fold between 10 days and 1 month p.i, followed by a stable number of labeled RGCs until 9 months p.i. This illustrates that the total pool of eGFP^+^ cells is an important factor when analyzing fate-mapping studies [45].

Until now, most genetic fate-mapping studies have been carried out using tamoxifen-inducible transgenic mice like Nestin-CreER^T2^, GLAST-CreER^T2^ or GFAP-CreER^T2^ mice that are crossed with a floxed reporter line [40,52,53]. The disadvantage of the latter two is that in addition to RGCs, also mature astrocytes are labeled upon tamoxifen induction. Fate mapping based on Cre-Flex LV vector injections in Nestin-Cre mice offers clear advantages in comparison to fate mapping based on crossing Nestin-CreER^T2^ mice with a floxed reporter line. Until now, nine different Nestin-CreER^T2^ lines have been developed in which neurogenesis in either the SVZ or the DG has been analyzed. A recent study now compared three of these lines showing that both the efficiency and specificity is remarkably different between those lines and is even affected by the choice of reporter line [54]. Importantly, the authors did not only find labeled cells in the SVZ and DG, but also substantial labeling outside the main neurogenic areas, e.g. in the cerebellum, cortex, striatum and even in mature granular neurons in the DG. On the contrary, local stereotactic injection of Cre-Flex LV vectors in transgenic Nestin-Cre mice adds an extra layer of spatial specificity, limiting transgene expression within the radius of viral vector spread. This spatial limitation might not only be useful for fate mapping purposes, but also when studying the effect of overexpression or downregulation of a protein of interest in the nestin^+^ RGCs. Overexpression of a protein of interest can be easily incorporated into the current Cre-Flex construct via an extra viral 2A sequence [38]. Additionally, we recently developed a new technology, based on the Cre-Flex strategy, for cell-type specific knockdown, which resulted in specific and functional knockdown both in cell culture and in mouse brain (Osorio et al., unpublished results). Alternatively, generation of double or triple transgenic mice is not only time-consuming, it will also automatically target both neurogenic regions. Moreover, in these mice it might be difficult to evaluate whether an observed effect on neurogenesis is direct, due to targeting of nestin^+^ RGCs, or indirect, due to unspecific targeting of e.g. cortical cells. Local stereotactic injection of Cre-Flex LV vectors provides additional spatial selectivity to address these questions. In inducible transgenic mice, manipulating the dose of tamoxifen can lead to either labeling of a whole population or either sparse labeling to study cells at the clonal level. In our present study, we injected a large volume (4 μL) of the Cre-Flex LV vectors into the DG of Nestin-Cre mice in order to achieve high labeling efficiency to be able to detect the labeled cell population with BLI. However, by injecting volumes in the nanoliter range, we believe that clonal analysis should be feasible with our Cre-Flex LV vectors. In comparison to cross-breeding Nestin-CreER^T2^ mice, our Cre-Flex LV approach is more labor-intensive, since stereotactic surgery is required for every single animal. In addition, these stereotactic injections induce a certain degree of injury, which might influence the experimental outcome. Also transgene expression requires some time delay, thereby precluding analysis at early time points such as 2 days after injection.

In conclusion, Cre-Flex LV technology allows to specifically label nestin^+^ RGCs in the DG of Nestin-Cre mice. We believe that this Cre-Flex LV technology will add extra value to the available toolbox in order to tackle the remaining unresolved questions regarding NSC identity, NSC behavior and the functional role and therapeutic potential of adult hippocampal neurogenesis.

## Supporting Information

S1 Table Absolute number of eGFP cells at different time points p.i. These data reflect the raw data shown in Fig 4A.
(XLSX)

S2 Table Proportion of different labeled cell types at different time points p.i. These data reflect the raw data shown in Fig 7A.
(XLSX)

S3 Table Absolute number of labeled cells within each population at different time points p.i. These data reflect the raw data shown in Fig 7B.
(XLSX)
